# Age, body composition parameters and glycaemic control contribute to trabecular bone score deterioration in acromegaly more than disease activity

**DOI:** 10.3389/fendo.2023.1197725

**Published:** 2023-08-21

**Authors:** Ivana Ságová, Marián Mokáň, Ingrid Tonhajzerová, Marianna Rončáková, Peter Vaňuga

**Affiliations:** ^1^ Department of Endocrinology, National Institute of Endocrinology and Diabetology, Ľubochňa, Slovakia; ^2^ Comenius University Jessenius Faculty of Medicine, 1st Department of Internal Medicine, University Hospital Martin, Martin, Slovakia; ^3^ Comenius University Jessenius Faculty of Medicine, Department of Physiology, Martin, Slovakia

**Keywords:** acromegaly, bone mineral density, dual-energy x-ray absorptiometry, insulin-like growth factor 1, trabecular bone score

## Abstract

**Introduction:**

Impairment of bone structure in patients with acromegaly (AP) varies independently of bone mineral density (BMD). Body composition parameters, which are altered in patients with acromegaly, are important determinants of bone strength.

**Purpose:**

The aim of this study was to examine BMD and lumbar trabecular bone score (TBS) by dual-energy X-ray absorptiometry (DXA) and to assess its relationship with disease activity, age, glucose metabolism, and body composition parameters.

**Methods:**

This cross-sectional prospective study involved 115 patients with acromegaly (70 F, 45 M) and 78 healthy controls (CON) (53 F, 25 M) matched for age, gender, and BMI. Bone mineral density, TBS and body composition parameters were measured using DXA.

**Results:**

AP presented with lower TBS compared to CON (1.2 ± 0.1 v 1.31 ± 0.1, P< 0.001). No significant correlation was observed between IGF-1/GH levels and TBS. Age, glycated haemoglobin, BMI, waist circumference, fat mass, and lean mass negatively correlated with TBS in both sexes. Multiple linear regression analysis of all these parameters revealed age and waist circumference as independent significant predictors of TBS in AP. We did not find difference in BMD (lumbar and femoral sites) between AP and CON nor between active and controlled AP. We observed negative correlation between age and BMD of the femoral neck and total hip (P < 0.001). Testosterone levels in males, BMI, waist circumference, fat mass, and lean mass positively correlated with BMD in AP, with stronger correlation between lean mass and BMD compared to fat mass.

**Conclusion:**

Patients with acromegaly have lower TBS than controls, confirming impaired bone microarchitecture in acromegaly regardless of BMD. Age, body composition parameters and glucose metabolism contribute to TBS deterioration in AP more than disease activity itself.

## Introduction

Acromegaly is a rare chronic progressive disease associated with multiple comorbidities and increased mortality. It is mainly caused by anterior pituitary tumours that secrete excessive amount of growth hormone (GH), resulting in overproduction of insulin-like growth factor 1 (IGF-1) (1). Long-term presence of elevated GH and IGF-1 levels, accompanying this disease, is associated with multiple complications, such as osteopathy, tissue hypertrophy and metabolic disorders. GH/IGF-1 overproduction in acromegaly leads to increased bone turnover with negative calcium balance (2, 3). Increased bone remodelling is likely due to IGF-1 induction of RANKL (receptor activator of nuclear factor kappa-β ligand) leading to enhanced osteoclastogenesis (4, 5). Data on bone mineral density (BMD) are conflicting. Some studies report both decreased and increased BMD in patients with active acromegaly (6, 7). GH/IGF-1 excess may cause abnormalities in trabecular and cortical bone architecture leading to decreased bone strength and increased risk of vertebral fracture irrespective of BMD (8–11). Bone quality in acromegaly depends not only on BMD but also on the microstructure of bone (8). Factors which can affect bone metabolism in acromegaly are presence of hypogonadism (with or without hyperprolactinemia), diabetes mellitus, age (9) or, potentially, body composition parameters. GH/IGF-1 overproduction stimulates proteosynthesis and increased lipolysis in adipose tissue. Active acromegaly is associated with decreased total body fat, and increased lean body mass and total body water (12, 13). Changes in body composition caused by IGF-1 overproduction could affect bone quality in acromegaly. Dual-energy X-ray absorptiometry (DXA), generally used for measuring BMD, plays a major role in estimating fracture risk and it is also very important in evaluating body composition (14). It has been established that in acromegaly bone strength and quality depend not only on BMD but also on skeletal characteristics (bone size and geometry, microarchitecture of trabecular and cortical compartments, microdamages, bone turnover, composition of the mineralized protein matrix) that are not measured by DXA (15). Trabecular bone score (TBS) is a novel method, which indirectly assess trabecular microstructure based on DXA image of the lumbar spine. TBS is useful, especially in secondary osteoporosis, in conditions such as glucocorticoid induced osteoporosis, diabetes mellitus, GH deficiency, and hypercortisolism (16).

This study had two main aims. One was to analyse bone density and quality, measured by lumbar TBS, in patients with acromegaly compared with sex, age and BMI matched controls. The other was to establish any possible relationship between BMD/TBS in patients with acromegaly and 1) disease activity, 2) body composition parameters 3) age, and 4) glucose metabolism.

## Materials and methods

### Patients

The study population consisted of 115 patients with acromegaly (AP) (70 F, 45 M) and 78 healthy volunteers as controls (CON) (53 F, 25 M) matched for sex, age and BMI.

Patients with acromegaly:

Patients with presence of acromegaly. The diagnosis was based on the established criteria: GH > 1µg/l during oral glucose tolerance test (oGTT) before receiving any treatments, IGF-1 levels above normal range for age and sex, and presence of a pituitary adenoma at magnetic resonance imaging.
Active acromegaly was diagnosed as: an increased IGF-1levels, random GH > 1µg/l and nadir GH ≥ 0.4 µg/l during oGTT (17).
No presence of: history of osteoporosis treatment, history of trauma, presence of diseases possibly leading to secondary osteoporosis (hypercortisolism, hyperthyroidism or thyrotoxicosis, hyperparathyroidism, malabsorption, chronic renal failure, rheumatoid arthritis, etc.), history of treatment that can impact bone structure (glucocorticoids, immunosuppressive drugs, etc.), history of cancer, presence of uncontrolled diabetes mellitus (HbA1c > 7.0%), or untreated anterior hypopituitarism.
In total, we excluded 24 patients with acromegaly.

Healthy control subjects matched for sex, age and BMI:

Subjects without acromegaly (normal IGF-1 values).
No presence of: primary or secondary osteoporosis, history of trauma or osteoporosis treatment.

We collected data regarding familial history of osteoporosis, lifestyle, smoking habits, alcohol intake, previous fractures, secondary osteoporosis and reproductive status. Females were considered eugonad when they had regular menstrual periods or were on sex hormone replacement therapy. Males were considered eugonad if total testosterone was over 7 nmol/l. For statistical analyses, we divided our patients with acromegaly into 2 subgroups: AP with active disease (aAP) and AP with controlled disease (cAP) based on disease control. We considered acromegaly as active when IGF-1 level was above the upper limit of normal reference range for age and sex, or there was lack of GH suppression during oGTT. Otherwise, we considered the disease as controlled, even when it was maintained by ongoing treatment.

Patients were also grouped according to WHO definition of obesity into two groups: ≥ 30 kg/m^2^ (obese) and < 30 kg/m^2^ (non - obese).

### Clinical examination

In all study subjects, we performed several anthropometric measurements. Height (cm) and weight (kg) were measured in light indoor clothing and without shoes. We used a wall mounted Harpenden stadiometer (Holtaim Ltd., UK) to measure standing height and a calibrated electronic scale (SECA 877, Germany) for body weight. BMI was calculated as weight in kilograms divided by square of height in meters (kg/m^2^). Waist circumference (WC) was measured just above the uppermost lateral border of the right ilium in standing position.

### Laboratory examinations

Venous blood samples were obtained between 07:00 and 08:00 am after overnight fasting. Pituitary hormones, serum phosphate and calcium, liver enzymes, creatinine, lipid profile, serum fasting glucose, insulin, and glycated haemoglobin were routinely measured at Alpha Medical Laboratory with standardized methods. We used American Diabetes Association Guidelines to diagnose glucose metabolism disorders (18). Insulin resistance was estimated by the Homeostasis Model Assessment (HOMA IR) using the following formula: fasting insulin (µU/l) x fasting glucose (mmol/l)/22.5 (18). IGF-1 and GH levels were measured using ECLIA chemiluminescent immunometric assay (Immulite 2000 assay, Siemens Healthcare Diagnostics Products Ltd., United Kingdom). Intraassay variability (CV) was for IGF-1 between 3.0 – 7.6% and for GH between 6.5 – 6.6%. All IGF-1 values were obtained with the same assay in the same laboratory and the diagnosis and subsequent monitoring were performed in a single laboratory and by the same method. Total procollagen 1 N-terminal peptide (P1NP), 25-hydroxyvitamin D, C-terminal telopeptide of type 1 collagen (CTX) and parathyroid hormone (PTH) were measured by Elecsys reagent kits (Roche Diagnostics, Switzerland).

### Dual-energy X-ray absorptiometry

BMD was measured in 3 places (lumbar spine (L1-L4), total hip and femoral neck at the posteroanterior position) using DXA Hologic (Horizon A, Bedford, MA). BMD was expressed in absolute values (g/cm^2^) as well as standard deviation (SD) from the peak bone mass (T-score) and the expected mass for the age-matched population (Z-score). In men over the age of 50 and postmenopausal women, osteoporosis was diagnosed when BMD T-score was ≤−2.5. Osteopenia was diagnosed as T-score between −1.0 and -2.5 SD. For men below the age of 50 and women before menopause, BMD was considered “below the expected range for age” as Z-score ≤−2.0 (19). TBS was measured on the same lumbar vertebrae as BMD using TBS iNsight version 2.1 software (Med-Imaps, Pessac, France), version 3.0.2. TBS values were classified into three categories as follows: ≤ 1.2 (fully degraded microarchitecture); 1.21 to 1.34 (partially degraded microarchitecture); and ≥ 1.35 (normal). We included subjects with a BMI range of 18 to 37 kg/m^2^. Body composition was determined by the same DXA as BMD by whole-body software version 13.6. Coefficient of variation was 0.78% for fat mass and 0.52% for lean mass. DXA was performed in all patients. The android region was defined by the iliac crest at the lower boundary and the upper boundary was calculated as 20% of the distance between the neck and the iliac crest. The gynoid region includes the upper thighs and hips.

### Statistical analyses

All the data were statistically analysed using IBM SPSS version 25 (IBM SPSS Statistics, IBM Corporation, IL, USA). The Shapiro–Wilk test was used to check the normality of the data. Continuous data are presented as mean and ± standard deviation (SD) and the categorical data as numbers and percentages. Inter-group comparisons were performed by either Student’s t-tests or Mann–Whitney tests, depending on normality distribution of the studied parameter. Categorical variables were compared by the chi-square test. Univariate analyses and multiple regressions (Enter method) were performed to investigate possible correlations between TBS and the significant parameters. In patients with acromegaly, selected variables (age, waist circumference, BMI, HbA1c, lean mass, and fat mass as inputs, i.e. independent variables) were entered into univariate linear regression models and later multiple linear regression model (Enter method) with TBS as output (dependent variable). P ≤ 0.05 was deemed statistically significant.

## Results

A total of 115 AP (70 F, 45 M) were included in the study. The mean age of AP at entering the study was 54 ± 12 years. The age-, sex- and BMI-matched controls consisted of 78 healthy volunteers (53 F, 25 M) with the mean age of 57 years ± 10 years. Baseline characteristics of all subjects are summarised in **Table 1**.

**Table 1:** Baseline characteristics of patients with acromegaly and control group, subgroups of patients with acromegaly.

Characteristics	AP (n=115)	Healthy controls (n=78)	AP vs. healthy controls P-value	Active AP (n=72)	Controlled AP (n=43)	Active AP vs. controlled P-value	Obese AP (n=51)	Non-obese AP (n=64)	Obese AP vs. non-obese P-value
Gender (M/F)	45/70	25/53	NS	30/42	15/28	NS	24/27	21/43	NS
Disease duration (year)	9±8.7	–	–	5.8±8.7	11.8±9.2	< 0.001	7.9±8.9	8.2±8.6	NS
Age at the time of study (year)	54±12	57±10	NS	53±11	57±12	NS	54±12	55±11	NS
Smoking history (%)	20%	16%	NS	22%	19%	NS	21%	18%	NS
Hypogonadism + menopause (%)	57%	55.12%	NS	56%	58%	NS	63%	59%	NS
Baseline GH (ng/ml)	3.8±6.5	0.3±0.2	< 0.001	5.4±7.8	1.2±0.9	< 0.001	4.2±6.5	3.6±6.6	NS
IGF-1 (ng/ml)	320±256	133±38	< 0.001	433±264	131±41	< 0.001	358±259	290±252	NS
IGF-1/ULN	1.44±1.16	0.59±0.15	< 0.001	1.96±1.2	0.59±0.2	< 0.001	1.62±1.19	1.3±1.13	NS
TSH (mIU/l)	1.8±2.9	2.3±1.3	NS	1.9±3.3	1.5±2.3	NS	1.7±3.5	1.8±2.4	NS
Testosterone males (nmol/l)	12.2±6.4	15.1±6.5	NS	11.4±5.6	13.8±7.8	NS	12.3±5.3	14.3±7	NS
FSH (IU/l)	21.3±24.7	35.5±31.2	< 0.001	21.3±23.7	21.4±24.9	NS	17.1±23.4	24.7±25.3	NS
LH (IU/l)	9±10.4	15.9±13	< 0.001	9.2±10	8.7±11.2	NS	7.1±9.6	10.5±10.8	NS
Prolactin (mIU/l)	166±139	246±115	< 0.001	191±162	126±75	0.015	159±155	172±126	NS
ACTH (ng/l)	21.2±11.4	15.9±7.1	< 0.001	23.2±12.3	17.8±8.8	0.007	21.1±10.7	21.3±12	NS
Plasma cortisol (nmol/l)	397±146	423±124	NS	396±136	433±156	NS	382±137	409±152	NS
Therapy – all patients with acromegaly									
Treatment naïve	27								
Surgery	72								
SSA only	43								
SSA + PEG	16								
SSA + CAB	2								
Radiotherapy	38								

Data is presented as mean ± standard deviation (SD) and as percentage.

Level of significance was set at p ≤ 0.05.

ns, not statistically significant.

ACTH, adrenocorticotrophic hormone; AP, patients with acromegaly; CAB, cabergoline; FSH, follicle - stimulating hormone; GH, growth hormone; IGF-1, insulin-like-growth factor 1; LH, luteinizing hormone; PEG, pegvisomant; SSA, somatostatin analogues; TSH, thyroid stimulating hormone; ULN, upper limit of the normal range.

### Comparison between patients with acromegaly and healthy controls (laboratory and body-composition parameters)

Serum levels of GH and IGF-1 were significantly higher in AP than in CON (**Table 1**). There was no statistically significant difference in levels of TSH, plasma cortisol, testosterone (males), but levels of ACTH, FSH, LH and PRL were lower in AP compared to CON (**Table 1**). There was no statistically significant difference in prevalence of hypogonadism/menopause. Serum level of glycated haemoglobin was significantly higher in AP compared with CON. We did not find any significant difference in BMI, waist circumference and fat mass between both groups (**Table 2**). Compared to CON, AP presented with higher lean mass (57.1 ± 14.9 kg *vs* 50.5 ± 11.9 kg, P< 0.001) (**Table 2**). There was no statistically significant difference in parameters of calcium-phosphate metabolism and CTX between AP and CON. P1NP was higher in AP compared to CON.

**Table 2:** Metabolic parameters, anthropometric and body composition parameters.

Characteristics	AP (n=115)	Healthy controls (n=78)	AP vs. healthy controls P-value	Active AP (n=72)	Controlled AP (n=43)	Active AP vs. controlled P-value	Obese AP (n=51)	Non-obese AP (n=64)	Obese AP vs. non-obese P-value
Fasting plasma glucose (mmol/l)	5.63±0.99	5.6±6.26	NS	5.79±1.08	5.36±0.77	0.025	5.98±1.16	5.35±0.74	< 0.001
HOMA IR	3.14±3.49	3.05±5.84	NS	3.54±2.08	2.46±4.38	NS	4.19±4.53	2.3±2.03	0.003
Glycated haemoglobin (%)	5.62±0.5	5.37±0.38	< 0.001	5.66±0.25	5.55±0.47	NS	5.84±0.44	5.45±0.47	< 0.001
Height (cm)	170±9	168±9	NS	172±9	171±9	NS	173±10	171±9	NS
Weight (kg)	88±19	84±18	NS	90±19	85±19	NS	103±14	76±13	< 0.001
Waist circumference (cm)	98±13	95±14	NS	102±12	98±13	NS	110±9	93±10	< 0.001
BMI (kg/m^2^)	29.6±5.2	28.9±6.7	NS	30.3±5.3	28.9±5	NS	34.3±3.3	25.9±3	< 0.001
Fat mass (kg)	31.4±9.3	31.8±10.2	NS	31.1±9.2	31.9±9.5	NS	38.1±7.6	26.1±6.8	< 0.001
Android fat mass (kg)	2.7±1.1	2.8±1.1	NS	2.7±1.1	2.7±1	NS	3.5±0.8	2±0.7	< 0.001
Gynoid fat mass (kg)	5.2±1.5	5.1±1.6	NS	5.1±1.5	5.4±1.5	NS	6.1±1.4	4.5±1.2	< 0.001
Trunk fat mass (kg)	14.9±5	15.6±5.4	NS	15.1±5.1	14.7±4.7	NS	18.4±3.6	11.9±3.5	< 0.001
Limbs fat mass (kg)	15.2±4.8	15±5.3	NS	14.8±4.5	15.9±5.3	NS	18.2±4.4	12.8±3.7	< 0.001
Lean mass (kg)	57.1±14.9	50.5±11.9	< 0.001	60.2±15.3	52±12.7	< 0.001	65.5±14.5	50.5±11.4	< 0.001
Android lean mass (kg)	4.6±1.2	4.3±1.1	0.036	4.3±1.2	4.3±1.1	0.015	5.4±1.1	4±1	< 0.001
Gynoid lean mass (kg)	9±2.3	8.1±1.9	0.008	9.4±2.3	8.2±2.1	0.011	10.3±2.2	7.9±1.8	< 0.001
Trunk lean mass (kg)	29.9±7.2	26.8±6.2	0.003	31.4±7.4	27.4±6.2	0.003	34.3±6.7	26.4±5.6	< 0.001
Limbs lean mass (kg)	25.8±7.8	22.5±5.9	< 0.001	27.3±8	23.3±6.7	0.009	29.7±8	22.7±5.9	< 0.001

Data is presented as mean ± standard deviation (SD).

Level of significance was set at p ≤ 0.05.

ns, not statistically significant.

AP, patients with acromegaly; BMI, body mass index; HOMA IR, homeostatic Model Assessment for Insulin Resistance.

### Comparison between active and controlled patients with acromegaly

Patients with acromegaly with active disease (aAP) presented with higher IGF-1 and GH levels compared patients with acromegaly with controlled disease (cAP) (P < 0.001). There was no statistically significant difference in levels of TSH, plasma cortisol, testosterone (males), FSH, and LH. However, levels of ACTH and PRL were higher in aAP compared to cAP (**Table 1**). There was no significant difference in levels of glycated haemoglobin, BMI, waist circumference and fat mass between the groups. Lean mass was significantly higher in aAP compared to cAP (**Table 2**). We did not find any difference in parameters of calcium-phosphate metabolism between aAP compared to cAP, but levels of P1NP and CTX were significantly higher in aAP compared with cAP.

### Comparison between obese and non-obese patients with acromegaly

We didn´t find any significant difference in levels of the pituitary hormones between obese (oAP) and non-obese patients with acromegaly (noAP) (**Table 1**). Level of fasting plasma glucose, glycated haemoglobin and insulin resistance (HOMA IR) were significantly higher in oAP compared with noAP. Compared to noAP, oAP presented with higher BMI, waist circumference, fat mass and lean mass (**Table 2**). There was no statistically significant difference in levels of calcium, phosphate, PTH, P1NP, and CTX between oAP and noAP. Level of the 25-hydroxyvitamin D was lower in oAP compared to noAP (**Table 3**).

**Table 3:** Biochemical parameters, TBS and bone mineral density.

Characteristics	AP (n=115)	Healthy controls (n=78)	AP vs. healthy controls P-value	Active AP (n=72)	Controlled AP (n=43)	Active AP vs. controlled P-value	Obese AP (n=51)	Non-obese AP (n=64)	Obese AP vs. non-obese P-value
Calcium (mmol/l)	2.4±0.1	2.35±0.1	NS	2.4±0.1	2.4±0.1	NS	2.4±0.1	2.4±0.1	NS
Phosphate (mmol/l)	1.2±0.2	1.1±0.2	NS	1.3±0.2	1.2±0.2	NS	1.3±0.2	1.2±0.3	NS
25-hydroxyvitamin D (nmol/l)	72±29	68±20	NS	68±29	79±27	NS	64±28	78±29	0.010
PTH (pmol/l)	6.4±3.6	7.4±5	NS	6±2.7	7.3±4.6	NS	6.6±3.3	6.3±3.8	NS
P1NP (pg/ml)	72±49	61±24	0.037	85±55	50±28	< 0.001	79±55	66±44	NS
CTX (µg/l)	0.6±0.4	0.5±0.2	NS	0.7±0.5	0.4±0.2	< 0.001	0.6±0.4	0.6±0.5	NS
**Lumbar spine TBS**	1.2±0.1	1.31±0.1	< 0.001	1.2±0.1	1.2±0.1	NS	1.1±0.1	1.2±0.1	< 0.001
Lumbar BMD (L1-L4)									
BMD (g/cm^2^)	1.012±0.159	1.014±0.154	NS	1.007±0.17	1.019±0.142	NS	1.046±0.171	0.984±0.145	0.039
T-score	- 0.5±1.4	- 0.6±1.4	NS	- 0.3±1.4	- 0.1±1.3	NS	- 0.1±1.5	- 0.8±1.4	0.047
Z-score	- 0.3±1.4	- 0.1±1.3	NS	- 0.3±1.4	- 0.3±1.4	NS	- 0.2±1.7	- 0.3±1.1	NS
Femoral neck BMD									
BMD (g/cm^2^)	0.842±0.147	0.822±0.144	NS	0.858±0.15	0.815±0.139	NS	0.9±0.162	0.8±0.119	< 0.001
T-score	- 0.5±1.1	- 0.8±1.1	NS	- 0.4±1.1	- 0.7±1	NS	- 0.1±1.2	- 0.8±0.9	0.005
Z-score	0.4±1.2	0±1.3	NS	0.4±1.2	0.3±1.2	NS	0.8±1.3	0.1±0.9	NS
Total hip BMD									
BMD (g/cm^2^)	1.007±0.151	1.007±0.154	NS	1.024±0.151	0.977±0.148	NS	1.074±0.149	0.957±0.134	< 0.001
T-score	0.1±1.1	0.2±1.1	NS	0.2±1.1	- 0.1±0.9	NS	0.6±1.1	- 0.3±0.9	0.001
Z-score	0.6±1	0.6±1.2	NS	0.7±0.9	0.4±1.1	NS	0.9±1	0.3±0.8	NS

Data is presented as mean ± standard deviation (SD).

Level of significance was set at p ≤ 0.05.

ns, not statistically significant.

AP, patients with acromegaly; BMD, bone mineral density; BMI, body mass index; CTX, β-C terminal telopeptide of type 1 collagen; HOMA IR, homeostatic Model Assessment for Insulin Resistance; P1NP, total amino-terminal peptide of procollagen type I; PTH, parathyroid hormone; TBS, trabecular bone score.

### Relationships between TBS/BMD and activity of acromegaly, body-composition parameters in patients with acromegaly

We found significant difference in TBS between AP and CON (1.2 ± 0.1 *vs* 1.31 ± 0.1, P< 0.001) (**Table 3**). There was no statistically significant difference in TBS between active and controlled AP. No significant correlation was observed between IGF-1/GH levels and TBS (**Table 4**). Obese AP presented with lower TBS compared with non-obese AP (1.1 ± 0.1 *vs* 1.2 ± 0.1, P< 0.001) (**Table 3**). BMI and waist circumference strongly negatively correlated with TBS in both sexes (P < 0.001). Significant negative correlation was found between TBS and fat mass in both sexes (P < 0.001). Trunk fat mass and android fat mass significantly negatively correlated with TBS in both sexes (P < 0.001) (**Table 4**). We found a negative correlation of lean mass, trunk lean mass and android lean mass with TBS in both sexes (**Table 4**). We didn´t find any correlations between limb lean mass, gynoid lean mass and TBS (**Table 4**). We found negative correlation between AP age and TBS (P < 0.001) (**Table 4**). TBS negatively correlated with plasma glucose (R = - 0.279, P = 0.003), HOMA IR (R = - 0.225, P = 0.016) and levels of glycated haemoglobin (R = - 0.272, P = 0.003). TBS positively correlated with lumbar spine BMD in both females (R = 0.332, P = 0.005) and in males (R = 0.305, P = 0.017). Age, glycated haemoglobin, BMI, waist circumference, fat mass and lean mass were significant negative predictors of TBS values at the univariate linear regression analysis. Interestingly, when considering all these parameters in the multiple regression model, only age and waist circumference were independent significant TBS predictors (**Table 5**).

**Table 4:** Correlation analyses.

	Age (years)	GH (ng/ml)	IGF-1 (ng/ml)	IGF1/ULN	Testosterone in males (nmol/l)	Glycated hemoglobin (%)	BMI (kg/m^2^)	Waist circumference (cm)	Fat mass (kg)	Trunk fat mass (kg)	Limbs fat mass (kg)	Android fat mass (kg)	Gynoid fat mass (kg)	Lean mass (kg)	Trunk lean mass (kg)	Limbs lean mass (kg)	Android lean mass (kg)	Gynoid lean mass (kg)
**TBS** **all AP**	R = - 0.376 P < 0.001	NS	R = -0.203 P = 0.097	NS	–	R = -0.272 P = 0.003	R = -0.505 P < 0.001	R = -0.567 P < 0.001	R = -0.459 P < 0.001	R = -0.466 P < 0.001	R = -0.343 P = 0.011	R = -0.487 P < 0.001	R = -0.351 P=0.010	R = -0.223 P = 0.017	R = -0.246 P = 0.008	NS	R = -0.343 P < 0.001	NS
**TBS** **Males**	R = - 0.348 P < 0.001	NS	NS	NS	NS	R = -0.375 P = 0.010	R = -0.577 P < 0.001	R = -0.537 P < 0.001	R = -0.618 P < 0.001	R = -0.610 P < 0.001	R = -0.328 P = 0.019	R = -0.603 P < 0.001	R = -0.384 P = 0.009	R = -0.374 P = 0.011	R = -0.26 P = 0.004	NS	R = -0.505 P < 0.001	NS
**TBS** **Females**	R = - 0.453 P < 0.001	NS	NS	NS	–	R = -0.315 P = 0.008	R = -0.481 P < 0.001	R = -0.580 P < 0.001	R = -0.393 P < 0.001	R = -0.397 P < 0.001	R = -0.393 P =0.003	R = -0.394 P < 0.001	R = -0.262 P = 0.008	R = -0.311 P = 0.009	R = -0.332 P = 0.005	NS	R = -0.414 P < 0.001	NS
**Lumbar BMD (L1-L4) (g/cm^2^) all AP**	NS	NS	NS	NS	R = 0.259 P = 0.006	NS	R = 0.233 P = 0.012	R = 0.242 P = 0.009	R= 0.185 P = 0.048	R = 0.185 P = 0.048	NS	R = 0.186 P = 0.047	NS	R = 0.235 P = 0.011	R = 0.351 P < 0.001	R = 0.335 P < 0.001	R = 0.233 P = 0.012	R = 0.264 P = 0.004
**Femoral neck BMD (g/cm^2^) all AP**	R = - 0.356 P < 0.001	NS	NS	NS	R = 0.338 P < 0.001	NS	R = 0.415 P < 0.001	R = 0.431 P < 0.001	R = 0.209 P = 0.028	R = 0.201 P = 0.034	R = 0.188 P = 0.048	R = 0.236 P = 0.013	NS	R = 0.616 P < 0.001	R = 0.615 P < 0.001	R = 0.614 P < 0.001	R = 0.579 P < 0.001	R = 0.602 P < 0.001
**Total hip BMD** **(g/cm^2^) all AP**	R = - 0.322 P < 0.001	NS	NS	NS	R = 0.441 P < 0.001	NS	R = 0.497 P < 0.001	R = 0.508 P < 0.001	R = 0.240 P = 0.011	R = 0.273 P = 0.004	NS	R = 0.309 P = 0.001	NS	R = 0.668 P < 0.001	R = 0.664 P < 0.001	R = 0.662 P < 0.001	R = 0.618 P < 0.001	R = 0.663 P < 0.001

Level of significance was set at p ≤ 0.05.

ns, not statistically significant.

AP, patients with acromegaly; BMD, bone mineral density; BMI, body mass index; GH, growth hormone; IGF-1, insulin like growth factor -1; BMI, body mass index; TBS, trabecular bone score; ULN, upper limit of the normal range.

**Table 5:** Univariate and multiple linear regressions evaluating the main predictor of TBS in patients with acromegaly.

Variable	Univariate Analysis				Multiple Analysis			
	Adjusted R^2^	B	β	P-value	Adjusted R^2^	B	β	P-value
**Age**	0.113	-0.004	-0.348	**< 0.001**	–	-0.004	-0.315	**0.001**
**Glycated haemoglobin**	0.066	-0.075	-0.272	**0.003**	–	-0.005	-0.020	0.817
**BMI**	0.248	-0.013	-0.505	**< 0.001**	–	-0.007	-0.259	0.177
**Waist circumference**	0.282	-0.006	-0.537	**< 0.001**	–	-0.004	-0.379	**0.036**
**Fat mass**	0.204	-0.007	-0.459	**< 0.001**	–	0.001	0.056	0.732
**Lean mass**	0.041	-0.002	-0.223	**0.017**	–	0.001	0.061	0.688
**All variables**					0.377			

Level of significance was set at p ≤ 0.05.

ns, not statistically significant.

BMI, body mass index; B, unstandardized B; β, standardized coefficient β.

Bold values are statistically significant correlations.

We found no significant difference in all BMD (lumbar spine, femoral neck, total hip) neither between AP and CON nor between active and controlled AP (**Table 3**). We didn´t find correlation between levels of the IGF-1/GH and all BMD (**Table 4**). We observed negative correlation between age and BMD of the femoral neck and total hip (P < 0.001). Testosterone in males positively correlated with all BMD. We didn´t find correlation between glycated haemoglobin and any BMD. All BMD was higher in oAP compared to noAP (**Table 3**). We confirmed positive correlation between BMI, waist circumference, fat mass, lean mass and all BMD (**Table 4**). We did not find correlation between gynoid fat mass and all BMD. Limb fat mass positively correlated only with BMD of the femoral neck. Lean mass more strongly positively correlated with BMD in patients with acromegaly compare to fat mass.

## Discussion

This prospective cross-sectional study on a large number of patients with acromegaly analysed bone density and quality, as measured by lumbar TBS, in patients with acromegaly and assessed their relationship with disease activity, patient´s age, glucose metabolism and body composition parameters. To our knowledge this is the first study on patients with acromegaly investigating the impact of body composition parameters on both BMD and TBS using DXA scan.

Bone resorption and formation are coupled processes which maintain bone homeostasis and are involved with both GH and IGF-1 (2, 20, 21). In acromegaly, chronic overproduction of GH/IGF-1 leads to increased bone turnover and negative calcium balance (2, 3). Increased bone resorption associated with GH renal effects induces bone loss and skeletal fragility (2). Patients with active acromegaly exhibit up to eight-fold-increased fragility fractures, however, it is due to compromised bone quality rather than quantity (11). Bone quality in acromegaly depends not only on BMD and bone turnover, but also on bone microstructure (8). Trabecular bones are more prone to GH overproduction than cortical bones, which leads to impairment of its structural integrity (22). Therefore, in patients with acromegaly, fractures occur even with normal or slightly decreased BMD (23–25).

In our study, TBS values in patients with acromegaly were significantly lower compared to the controls, without significant differences in BMD values at lumbar and femoral sites between the groups. This finding is consistent with previous results reported by other authors (22, 26–29). The effect of disease control on bone metabolism in acromegaly is still unclear. Hong et al. demonstrated a significantly decreased TBS in patients with active acromegaly compared to controls, although BMD was similar in both groups (30). Calatayud et al. revealed higher TBS values in patients with post-surgical remission (31). Godang K et al. described decreased TBS values one year after surgery, although associated with increased BMD levels (32). Recently, Sala et al. and Jawiarczyk-Przybylowska et al. confirmed decreased TBS values in AP in comparison to controls, despite no difference in BMD among the groups (22, 26). We did not find any statistically significant difference in TBS and BMD (lumbar and femoral sites) between active and controlled AP. Active AP presented with increased P1NP and CTX levels, which could indicate accelerated bone turnover. There was no significant correlation between GH/IGF-1 and BMD values in AP nor between GH/IGF-1values and TBS. GH and IGF-1 did not show as major TBS determinants. Of note: at the time of TBS/BMD examinations about a third of our patients with acromegaly had achieved biochemical control of acromegaly and the average disease history was 9 ± 8.7 years.

Changes in body composition are also typical in acromegaly. Overproduction of GH/IGF-I leads to increased proteosynthesis and lipolysis in adipose tissue (33). Active acromegaly is associated with increased lean body mass, decreased total body fat, and increased total body water (13, 33, 34). These changes are associated with severity of the disease and GH/IGF-I levels, and we are able to normalise them with a successful treatment (35). We investigated the impact of body composition on both TBS and BMD values. AP presented with higher lean mass compared to controls, with no other differences in body composition parameters.

We also compared obese and non-obese AP. Obese AP presented with higher BMI, WC, fat mass, lean mass, fasting plasma glucose, HbA1C, insulin resistance, and lower level of 25-hydroxyvitamin D. BMD (lumbar and femoral sites) was higher in obese compared to non-obese AP. We confirmed positive correlation between BMI, WC, fat mass, lean mass and BMD (lumbar and femoral sites) in both groups. Higher BMD in obese subjects, reported by several authors, can be attributed to the mechanical effect of body weight on bone (36–39), which is stronger by lean than by fat mass (40–42). In our study, lean mass more strongly positively correlated with BMD in AP compared to fat mass. The skeletal muscle is one of the major components of lean mass. Lean mass increase favours increase of BMD and improved geometry and bone modelling. Weight bearing effect on bone is mainly exerted on lower limbs (43). Santos et al. confirmed a direct relationship between increased lean mass and bone density (total bone density, femur and spine), even though sarcopenic obesity causes osteoporosis (44). Recent research suggests that the impact of muscles on BMD goes beyond mechanics and it is also influenced by their secretory activity; therefore muscles, with their production of myokines, can be considered as an endocrine organ (45, 46).

In general, obese patients have a significantly poor bone microstructure compare to non-obese patients (39). In our study, TBS was significantly lower in obese AP compared to non-obese AP. Obesity negatively affected TBS, despite unchanged BMD. We found BMI significantly negatively correlating with TBS in AP in both sexes. In other studies, correlation between TBS and BMI varies significantly, which could be related to different study population and research designs (retrospective, prospective or cross-sectional), and by various DXA machines used (Hologic DXA/Lunar DXA). In a study of 1474 postmenopausal Korean women, Kim et al. confirmed a positive correlation between TBS and BMI (Lunar DXA) (47). However, a study of 250 Italian men and women by Bazzocchi et al. reported no correlation (Lunar DXA) (48). Several studies demonstrated that lower TBS is strongly related to increased BMI (Hologic DXA) (46, 49–51). McCloskey et al. performed a large meta-analysis of men and women of different ethnic origins, using both Hologic and Lunar DXA. They reported an overall weaker inverse correlation between TBS and BMI (52). Recently, Calatayud et al. confirmed lower TBS in patients with acromegaly with higher BMI (31).

In our study, fat mass negatively correlated with TBS in both sexes, which explains higher fracture risk in obesity. We found significant negative correlation between TBS and WC in both sexes, which confirms previous findings that the effect of WC on TBS is more pronounced than that of BMI (45).

The influence of adiposity on skeletal microarchitecture may depend on fat distribution. In our study, trunk fat mass and android fat mass more strongly negatively correlated with TBS in both sexes compared to gynoid and lower limb fat. This confirms previous findings that central obesity is more damaging to health (including bones) than lower body obesity (53). Abdominal obesity, more common in males, is associated with higher insulin resistance as well as systematic inflammation and oxidative stress (54, 55). Increased circulating inflammatory cytokines (tumour necrosis factor α, interleukin 1,12,17,18,33 and interferons), altered levels of bone-regulating hormones (adipokines), and increased aromatization of androgens have adverse effect on bone metabolism (54–56). Impaired glucose tolerance or overt DM are well recognized as comorbidities in acromegaly that can influence TBS (57). In our study, TBS negatively correlated with plasma glucose, HOMA IR, and levels of glycated haemoglobin in AP in both sexes. Low TBS in patients with DM may be caused by accumulation of advanced glycosylation end-products (AGEs) in bones (58). The accumulation of cross-links that are caused by non-enzymatic glycation may lead to reduced bone turnover as a result of altered responses of osteoblasts and osteoclasts to AGEs (58). Both low bone turnover and glycated collagen matrix may lead to brittle bones, regardless of BMD (58).

In our study, there was no correlation of limb and gynoid lean mass with TBS. Lean mass, trunk lean mass and android lean mass negatively correlated with TBS in AP in both sexes. The negative correlation could be explained by the amount of different tissues in the trunk, as the amount of lean trunk mass on average doubles that of the average trunk fat mass (59). In active acromegaly, acceleration effect of excess GH/IGF-I on anabolism results in additional increase in lean mass, including in trunk (60). Another cause could be the artefactual effect of the regional presence of the soft tissue on the DXA scan, which could affect TBS values. The question remains whether the association between TBS and incidence of fractures in acromegaly becomes weaker with increased BMI or trunk lean mass. To establish this, further large studies or meta-analyses are necessary.

Our findings of significant inverse correlation between age and TBS values confirmed previous research (61, 62). The annual rate of TBS loss has been reported to be accelerated after 65 years of age (62). Dufour et al. in a study of 5,942 women, reported a linear decrease of 14.5% in lumbar TBS values in women over the age of 40 (61).

In our study, univariate analyses revealed age, glycated haemoglobin, BMI, waist circumference, fat mass, and lean mass to be significant negative predictors of TBS values. Interestingly, multiple regression analysis of all these parameters found only age and waist circumference as independent significant TBS predictors in acromegaly.

## Conclusion

We confirmed lower TBS values in patients with acromegaly compared to healthy controls and no significant differences in BMD values at lumbar and femoral sites between the groups. The findings suggest that increased BMI, WC, lean and fat mass had a positive effect on BMD in acromegaly merely through mechanical loading. However, increased BMI, WC, fat mass, lean mass, age, and impaired glucose metabolism negatively contribute to TBS more than activity of acromegaly itself. The most important determinants of TBS values in acromegaly seems to be age and waist circumference.

We believe that our results have enhanced the understanding of pathology of impaired bone microstructure in acromegaly and that future prospective studies as well as meta-analyses further focusing on relationship between body composition and TBS in acromegaly would be useful.

### Strengths and limitations

The strengths of this study are the large number of patients with acromegaly and the implementation of different methods obtained from DXA examinations (BMD, TBS, body composition). This enabled us to assess the impact of multiple factors in a single study. To our knowledge, this is the first study that has investigated the correlations between body composition and TBS in patients with acromegaly. The limitations of our study include: (i) cross-sectional study design and the differences in the number of males and females (ii) various duration of acromegaly and the different types of applied treatment which could affect the values of TBS and BMD (iii) technical limitation of TBS examination (regional soft tissue by attenuating X-rays may have a noise effect on TBS leading to underestimated values). Further large studies are needed to confirm the effect of BMI and body composition parameters on the precision of TBS.

## Data availability statement

The original contributions presented in the study are included in the article/supplementary material. Further inquiries can be directed to the corresponding author.

## Ethics statement

The studies involving human participants were reviewed and approved by EC of the National Institute of Endocrinology and Diabetology in Ľubochňa, Slovakia. The patients/participants provided their written informed consent to participate in this study.

## Author contributions

IS, MM, IT, MR and PV contributed to conception and design of the study. IS prepared the manuscript. All authors contributed article and approved the submitted version.
